# Prevention and Treatment of Monkeypox: A Step-by-Step Guide for Healthcare Professionals and General Population

**DOI:** 10.7759/cureus.28230

**Published:** 2022-08-21

**Authors:** Lokesh Goyal, Kunal Ajmera, Ramesh Pandit, Trupti Pandit

**Affiliations:** 1 Hospital Medicine, Christus Spohn Hospital Corpus Christi - Shoreline, Corpus Christi, USA; 2 Hospital Medicine, Sentara Northern Virginia Medical Center, Woodbridge, USA; 3 Medicine, Independant Researcher, Philadelphia, USA; 4 Hospital Medicine, University of Pennsylvania, Chester County Hospital, Philadelphia, USA; 5 Pediatrics, Nemours Children's Hospital, Glen Mills, USA

**Keywords:** lgbtq+, msm, prevention, public policy, monkeypox treatment, monkeypox prevention, monkeypox transmission, monkeypox rash, monkeypox virus

## Abstract

The World Health Organization (WHO) recently declared the monkeypox virus a Public Health Emergency of International Concern (PHEIC). As the cases of the COVID-19 pandemic start to get under control, we have seen the monkeypox virus, found predominantly in Africa, spread in non-endemic countries worldwide. In the 1970s, after the smallpox virus eradication and the vaccine's discontinuation, the monkeypox virus infection started to gain attention. The first United States outbreak happened in 2003; since then, more sporadic cases of monkeypox have gained media attention. With cases spreading worldwide, without epidemiological links with outbreaks among men who have sex with men (MSM), it warrants urgent public health control measures to contain the spread of the monkeypox virus and investigate the underlying pathophysiology, including genetic modification of the virus. This review highlights the epidemiology, transmission, pathogenesis, clinical manifestation, diagnosis, prevention, and management of the current human monkeypox virus infection.

## Introduction and background

Monkeypox belongs to the same genus that causes smallpox. Many common viruses belong to this genus, including cowpox, horsepox, camelpox, and alaskapox. The variola virus is the most common and well-known member among all the viruses present in this genus. The World Health Organization (WHO) formally declared the eradication of smallpox in 1980 [1].

Even though the monkeypox virus is homologous to the smallpox virus, the mortality risk and contact transmission from monkeypox are substantially lower than that of smallpox [2,3]. However, the signs and symptoms, primarily the rash, are very similar to smallpox. In Africa, two distinct lineages of the monkeypox virus are identified, clade 1 and clade 2. Clade 1 is predominantly prevalent in Central Africa and the Congo Basin, whereas clade 2 is restricted to West Africa [2]. The clade 2 strain of monkeypox is linked to the current monkeypox pandemic, triggering a global public health emergency. However, a recent study shows that the circulating strain of the virus has had multiple mutations in its DNA genome, which raises the possibility that the circulating virus is changing for human adaptation, and contagion [3].

## Review

Methodology

A iterature search for monkeypox was performed using PubMed, ScienceDirect, and Google Scholar search engines. The search syntax included the following terms: "Monkeypox" and "origin," "spread", "symptoms", "prevention", "treatment", "vaccine", OR "risk to the healthcare worker". Four physician authors reviewed the literature, and the information is summarized in this article for simplicity of comprehension.

Epidemiology

**Figure 1:**
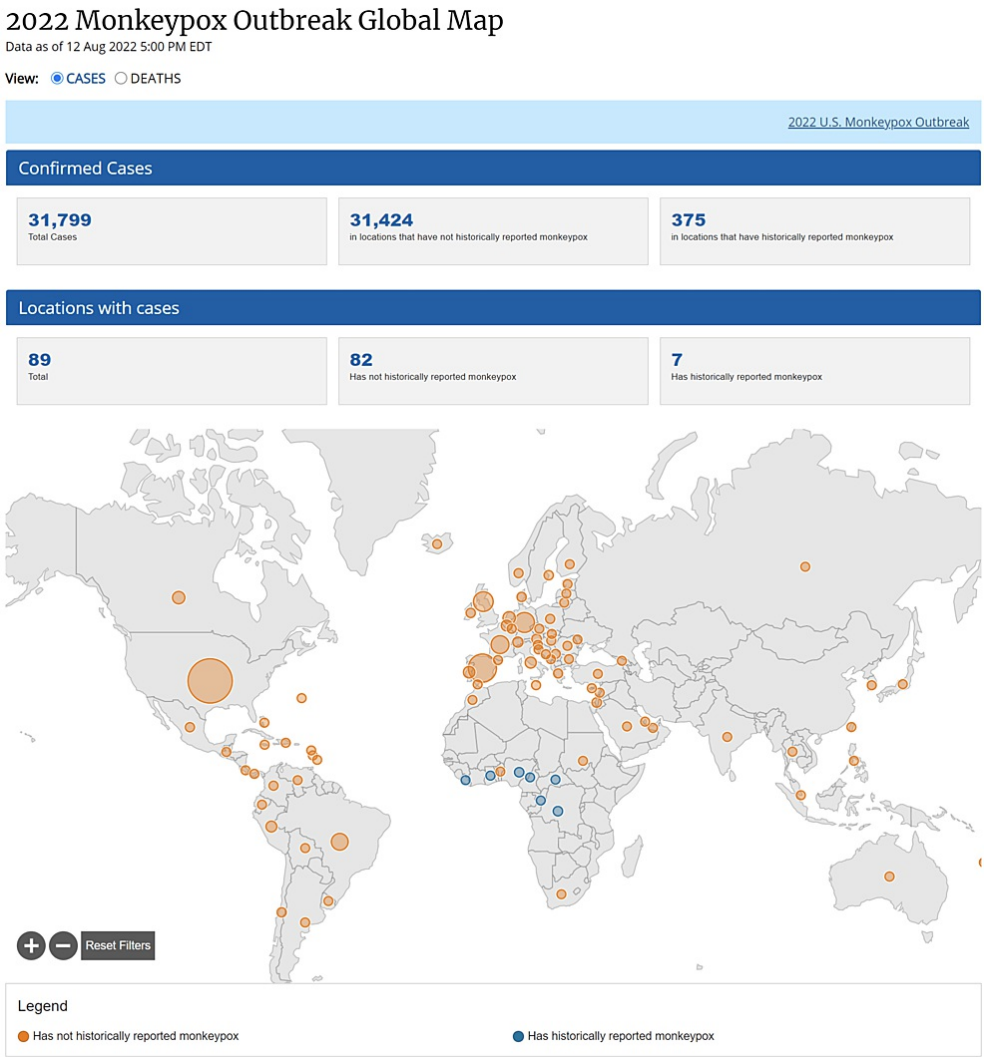
Monkeypox cases worldwide Monkeypox cases in the world as of August 12, 2022 [6]

**Figure 2:**
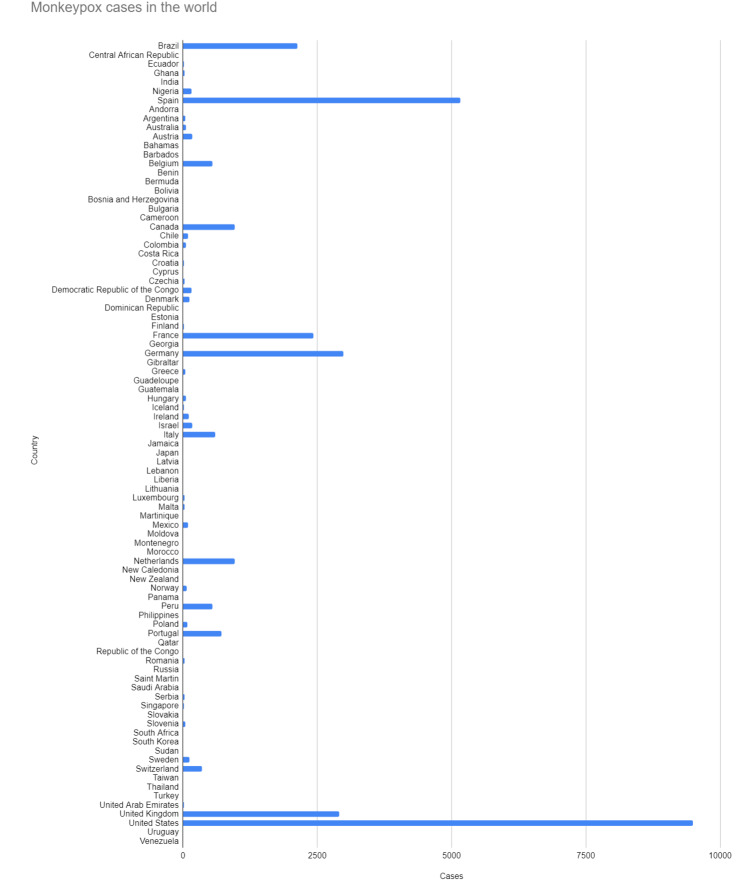
Number of confirmed monkeypox cases by country Number of confirmed cases by country [6]

Monkeypox was initially isolated in Denmark from monkeys used in the resource of poliovirus in 1959 [4]. Some sporadic monkey virus outbreaks have been seen in different animals since then. In the 1970s, researchers in Congo identified the first human case of monkeypox. Later, in the late 1970s, many instances of this virus were identified in Central and West Africa. Since the first monkeypox virus outbreak in the United States in 2003 [5], several sporadic cases have been observed in numerous non-endemic continents, including Europe and Asia. The current epidemic is widespread worldwide and exhibits person-to-person transmission (explained below). Figure 1 and Figure 2 show the total number of monkeypox cases worldwide as of August 12, 2022. The Center for Disease Control (CDC) publicly made the data available [6]. These figures highlight the countries most affected by the Monkeypox virus.

Immunization against smallpox also protects humans against the monkeypox virus; after the smallpox vaccination program was abandoned due to the disease's eradication, the WHO was concerned about greater susceptibility and incidence of monkeypox infection in the contemporary population. A survey conducted between 2005-2007 in the Democratic Republic of Congo showed that monkeypox infection in this country rose by 20-fold [7].

**Figure 3:**
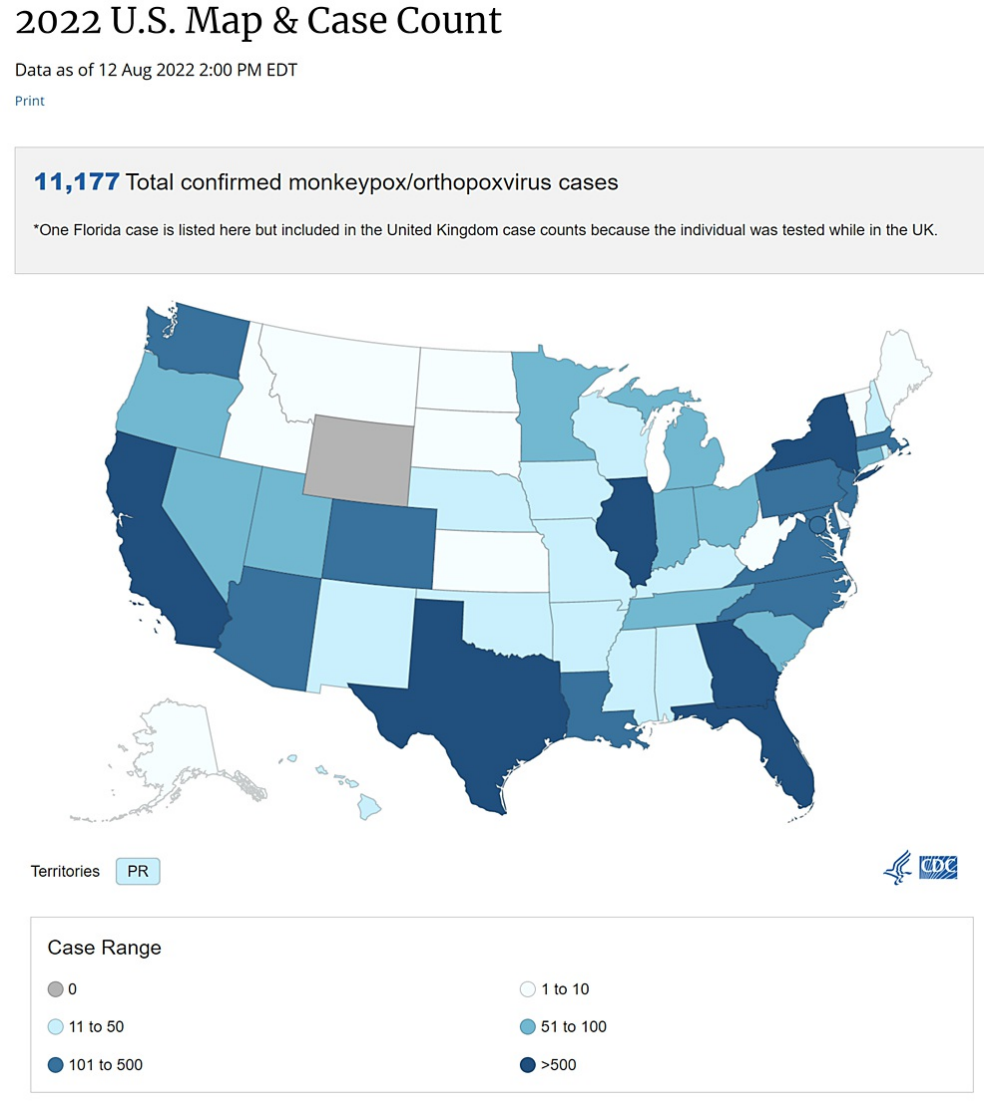
States affected by monkeypox States affected by Monkeypox virus in the United States [6]

**Figure 4:**
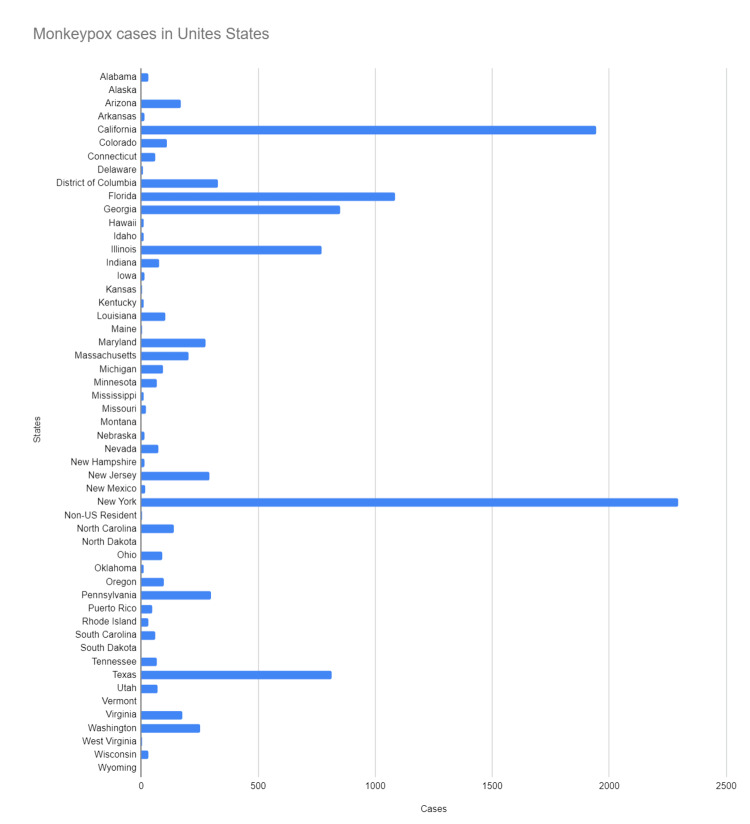
Breakdown of the total number of confirmed cases in the US by states Number of confirmed cases in the US by states [6]

**Figure 5:**
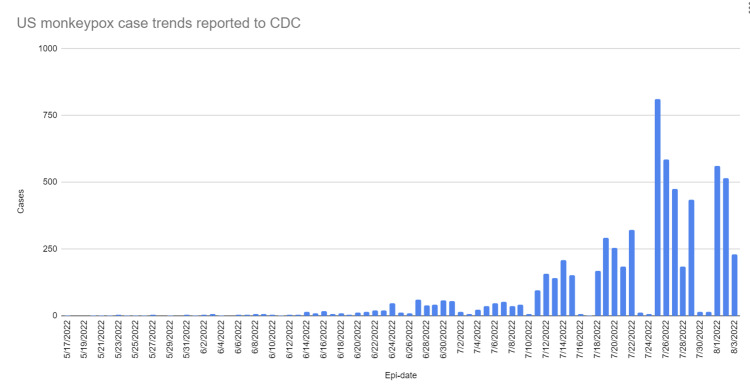
Trends in the current outbreak of monkeypox cases in the United States Trends in the current outbreak of monkeypox cases in the United States since May 17, 2022 [6]

Early May 2022 saw the isolation of the first cases of the current major monkeypox outbreak in Europe [8], and since then, cases have risen globally, including in non-endemic nations. WHO declared monkeypox a public health emergency of international concern on July 23, 2022 [9], and it is soon to reach pandemic levels. Figure 3 and Figure 4 show the total number of monkeypox cases in the United States and their breakdown by state. Figure 5 shows the rising trend of monkeypox cases in the United States [6]. 

The transmission of the monkeypox virus can happen in two ways

Animal to Human Transmission

During this transmission, humans come in contact with the virus via infected animals' body fluids or by an infected animal's bite. It can also be transmitted when consuming raw/minimally processed meat from an infected animal. This raw or minimally processed meat consumption is also known as bushmeat. The monkeypox virus in Africa is typically found in animals like squirrels, rats, and monkeys [10].

Human to Human Transmission

Human transmission can occur in different ways, which include direct contact, indirect contact, respiratory droplets, and vertical transmission [11-15].

Direct contact: Transmission occurs when a person comes in direct contact with the infection source, for example, infected body fluids or scabs. The ongoing outbreak of monkeypox in the world, especially in non-endemic countries, is thought to be due to the person's direct contact, mostly during sexual activities [11]. A non-infected person gets exposed to the infectious material from the skin of an infected person with close physical contact and therefore acquires this infection [11,12].

Indirect contact: Transmission occurs when materials like clothes or linens come in contact with infected material like bodily fluids. A non-infected person can then come in contact with these materials and get infected [13].

Respiratory transmission: Transmission occurs via respiratory secretions/droplets from coughing and sneezing [14].

Vertical transmission: Transmission occurs upon a fetus being exposed to the virus from her mother. The virus is known to cross the placenta and can lead to congenital monkeypox infection in newborn babies [15].

Future studies are required to better comprehend the role of virus transmission through genital fluids versus other secretions/forms [16]. Hence, labeling the disease as a sexually transmitted disease will lead to the underutilization of preventive efforts, which will move us closer to a pandemic scenario [16].

Pathogenesis

Monkeypox virus can be transmitted via animal or human contact, as described above. The virus enters the host, replicates at the entry site, and circulates via the lymphatic system. This leads to systemic infection, also known as primary viremia. The virus will now start replicating in the lymphoid organs and distant lymph nodes, resulting in infection of the epithelium and tertiary organs, producing mucosal and skin lesions, which results in the clinical manifestation of this virus [17].

Clinical manifestations

**Figure 6:**
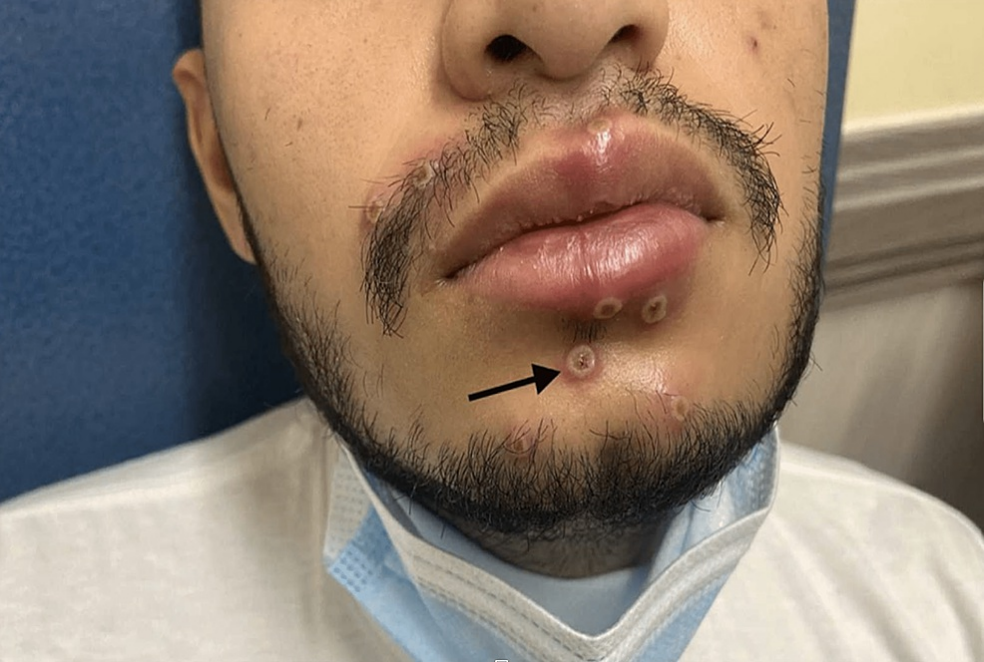
Monkeypox rash with pustules Reprinted from Ajmera et al. [18]

**Figure 7:**
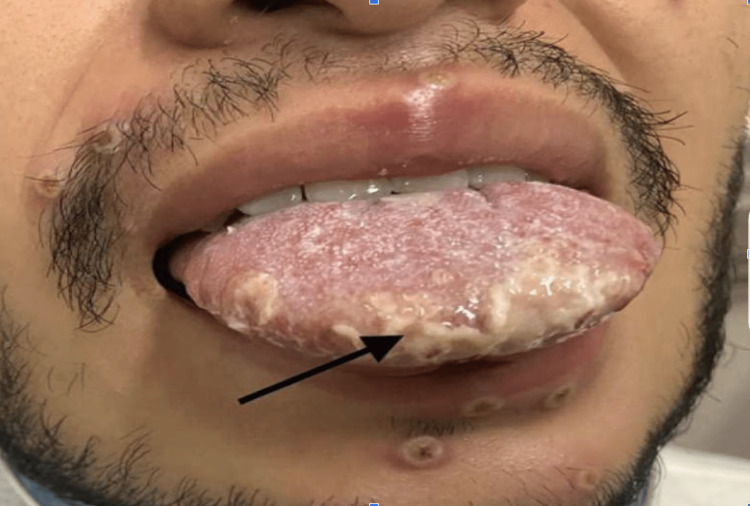
Monkeypox rash Reprinted from Ajmera et al. [18]

The incubation period for the monkeypox virus can range from five days to three weeks [17]. Monkeypox causes high-grade fever, chills, fatigue, lymphadenopathy, muscle pain, and vesicular eruptions with a rash. The prodromal phase is associated with all the symptoms but without a rash. The prodromal period lasts about four to five days. The rash typically appears after four to five days of fever and continues for two to three weeks (Figure 6, 7). 

However, some patients can get the rash without going through the prodromal phase [14]. The rash typically appears on the face but can also be found on the palms of hands and soles of the feet. Recent cases of monkeypox infection are associated with the involvement of the genitalia, anus, and oral mucous membranes [19,20]. The rash looks like a macule of around 2 to 5 mm in diameter. These macules/lesions will subsequently develop into vesicles and pustules. The lesions are very well-circumscribed, with central umbilication/depression on top of the lesion. These lesions will eventually crust over, dry, and fall off the skin in about one to two weeks. The rash during the developmental phase can be painful but mostly becomes itchy during the healing phase, i.e., during the formation of the dry crust [21]. The lab findings associated with monkeypox infection are nonspecific and include abnormal liver function enzymes, leukocytosis, and even thrombocytopenia [22]. The mortality risk associated with monkeypox varies depending upon which strain a person is infected with, i.e., clade 1 vs. clade 2. The current outbreak of monkeypox is similar to clad 2 strain which is less virulent than clad 1. So far, till July 2022, close to 14,000 cases of monkeypox worldwide have been reported, which include five deaths in African nations [23]. Immunocompromised status, complicated infections causing end-organ damage, children younger than eight years old, pregnant or breastfeeding women, and monkeypox infection in unusual places, including the eyes, are independent predictors of disease severity [24].

Diagnosis

Patients with typical rash and symptoms such as fever/chills should be suspected of having monkeypox infection, particularly if they have risk factors like recent travel to areas endemic for monkeypox. Additionally, patients who experience symptoms of sexually transmitted infections, such as genital rash, and who do not improve after receiving empiric treatment should be treated with a high degree of suspicion.

Laboratory evidence such as that of the predominance of the virus or IgM (immunoglobulin) antibodies must be present if monkeypox is suspected in the patient. Viral or serological testing can be used to determine this.

Viral testing includes a polymerase chain reaction (PCR) test performed on the infected lesion. A patient whose PCR test result shows positive *Orthopoxvirus *is suspected of having monkeypox infection per CDC guidelines for the 2022 outbreak of the monkey virus. Confirmation of the virus can be made when monkey virus DNA can be demonstrated by PCR.

Serological testing for the detection of monkeypox can be used if viral testing such as PCR is unavailable. The patient infected with the monkeypox virus will have elevated anti-*Orthopoxvirus *IgM & IgG antibodies, typically developing five to eight days after the rash [25].

Monkeypox virus-infected cells have a brick-like form on electron microscopy identical to variola or vaccinia virus-infected cells, as seen in Figure 3.

**Figure 8:**
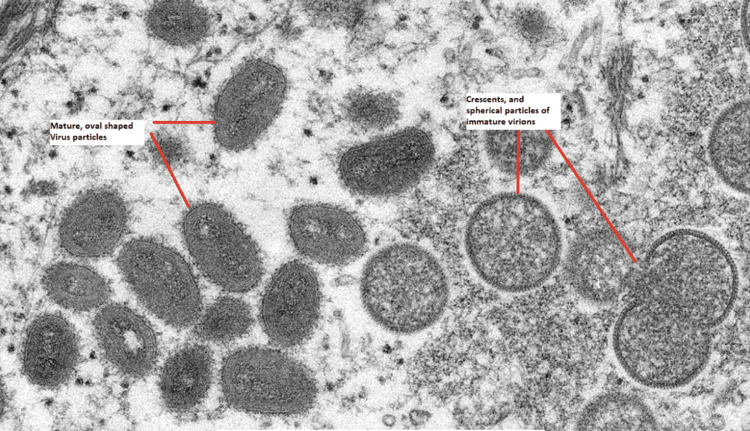
Monkeypox virus infected cells Image of monkeypox virus as seen from an electron microscopic in a human skin sample: CDC public health image library [26]

Treatment

The majority of monkeypox virus patients have mild symptoms and should make a full recovery without any professional attention. However, some patients could need hospitalization and supportive care due to nausea, vomiting, the potential for dehydration, or pain management. Antiviral medication is advised for those people who, as was previously noted, are at high risk of contracting a severe illness.

Multiple antiviral medications are available which can be used for the treatment of monkeypox. Some of these medications were developed for treating smallpox in animals but are expected to have the same activity against monkeypox [24,25]. 

Of all the antivirals available, tecovirimat is a treatment of choice for most cases. Patients with severe disease may also benefit from dual therapy of tecovirimat and cidofovir. Public health officials should be made aware when starting these treatments.

Tecovirimat

Tecovirimat is available in both an IV and oral form. This medication inhibits the VP37 protein present in *Orthopoxvirus *and therefore blocks the interaction of the virus with the host cell. This leads to inhibition of the infectious virus particles and prevents the host cell from getting infected. In 2018, the United States (US) approved tecovirimat to treat the smallpox virus [27]. Tecovirimat's dose depends upon the patient's weight and renal functions (on the IV); the treatment duration is 14 days. The only contraindication of tecovirimat, when given in IV form, is a severe renal impairment with creatinine clearance <30 mL/min. The oral form, however, has no contraindication with any form of renal function. Tecovirimat has not been studied in pregnant and breastfeeding females. It is not known whether tecovirimat is present in breast milk [27]. If treatment is indicated in a pregnant or breastfeeding patient infected with the monkeypox virus, tecovirimat should be considered the first line of treatment for these patients. As mentioned before, information is limited on whether tecovirimat affects the fetus in the reproductive development; however, animal studies in the past showed no specific fetal effects even though the dose used in the animal study was 23 times higher than the dose used in humans [28,10]. 

Cidofovir

This medication is effective against monkeypox in animal studies [27]. However, no significant clinical data exists on its efficacy toward monkeypox infection in humans. The mechanism of action of cidofovir includes converting cidofovir to its active metabolite called cidofovir diphosphonate. This cidofovir diphosphate then causes selective inhibition of viral DNA synthesis, suppressing virus replication. Cidofovir comes with a US boxed warning, including the possibility of severe nephrotoxicity. Severe acute renal failure resulting in dialysis can happen in as few as one to two doses of this medication. Renal functions must be monitored with each dose of cidofovir [29]. Patients who must be treated with this medication should also receive intravenous saline for rehydration and oral probenecid. Probenecid decreases the renal clearance of cidofovir by blocking the kidney's tubular secretions, reducing the incidence of nephrotoxicity [30].

Other US boxed warnings: This medication can be carcinogenic and teratogenic based on animal studies. Also, patients are at risk of neutropenia with this medication; therefore, the patient's neutrophil count must be monitored when receiving this medication. There is a risk for metabolic acidosis along with hepatic impairment and pancreatitis. Cidofovir should be avoided in pregnant females as it can be teratogenic; however, if use is warranted, systemic therapy should be avoided during the first trimester if possible [31]. It is not known whether cidofovir can pass into the breastmilk or not at this time. However, breastfeeding is not recommended because of serious adverse reactions possible with this medication [29,31].

Brincidofovir:

This medication is similar to cidofovir but likely has fewer side effects. This medication is a lipid conjugate that is converted to cidofovir intracellularly, which is then further converted to cidofovir diphosphonate, an active metabolite of cidofovir. Cidofovir diphosphate causes selective viral DNA inhibition, suppressing the virus application. Significant side effects include hepatotoxicity. Upon initiating this medication, patients with hepatic impairment should closely monitor their liver functions. [32] If a patient develops hepatic impairment during the treatment, consider discontinuation if alanine transaminase (ALT) remains consistently high, >10 times the upper limit of normal [33,34]. Pregnant females should avoid taking this medication as in utero exposure to brincidofovir may harm the fetus. It is unknown if brincidofovir can transfer into breastmilk; therefore, breastfeeding is not recommended when the patient is on this medication [34].

Trifluridine:

This medication can be used to treat and prevent corneal and conjunctival involvement in patients with monkeypox lesions involving the eye. This medication comes in topical antibiotic drops or ointment, which can be applied every four hours for seven to 10 days [35,36]. No dosage adjustment is needed for patients with hepatic and kidney impairment. Trifluridine mechanism of action involves interference with replication of the virus by inhibiting thymidylate synthetase, reducing the viral load.

Prevention

For prevention, a two-fold approach is needed. The first goal should be to promptly treat those infected and provide post-exposure management to minimize the onset of illness. The second goal will be at the public health and policy/ administrative level with increased funding and making diagnostic tests and vaccine availability a priority. 

Increased community awareness and wider surveillance of current cases and exposure is the first step toward preventing the spread [37,38]. There is a lack of evidence-based prevention methods, but we can utilize the knowledge learned from the COVID-19 pandemic [39]. From COVID-19, we learned that early diagnosis, quarantine for those with the infection, and open data sharing would be crucial for any community intervention to minimize the spread of any viral outbreaks [40]. 

Minimizing or avoiding stigmatization is needed to prevent discrimination and optimize disease response. Especially in the LGBTQ+ community and the public in general, aggressive educational campaigns regarding the transmission and spread will help minimize the stigma and, thereby, the hesitancy to seek help [41]. The stigmatization can also result in delays at the community, state, and even international levels, affecting political decision-making and resource allocation. In the past, Roess et al. noted educational activities to be effective in improving community-level knowledge and seeking professional advice [42]. Modifying the message delivery to match the current communication methods, including various forms of social media, should lead to similar results. The panic related to the current global health emergency, which is likely approaching the pandemic level, can be minimized with better education and unified messaging [39]. 

Key preventive measure is related to minimizing contact with the lesions, as close and prolonged contact appears to be the primary mode of transmission [11]. Social distancing from those with infection or post-exposure can also be considered useful, thereby minimizing possible respiratory transmission [12]. Avoiding sharing of beddings, towels, and clothes is needed [13].

Pre-exposure prophylaxis vaccine

The Advisory Committee on Immunization Practices (ACIP) recommends pre-exposure prophylaxis by using the modified vaccinia Ankara (MVA) vaccine for individuals who are at high risk due to occupational exposure to orthopoxvirus infections [43], for example, clinical laboratory personnel or scientists who are performing diagnostic testing for orthopoxviruses and designated response team members who are at risk of occupational exposure as well [44]. Experts are even discussing the use of pre-exposure prophylaxis MVA vaccine for high-risk patients. These individuals may be able to get vaccines once we have enough supplies of the MVA vaccine [45].

Post-exposure risk assessment

People should watch closely for symptoms for at least 21 days post-exposure with a monkeypox patient. If a person remains asymptomatic, they can resume their normal activities; however, if they start to experience symptoms, they should quarantine themselves and seek further medical attention from their primary care provider or the health department. There are three categories of risk of exposure [36].

High-Risk Exposure

CDC considers patients as high-risk exposure if they had: a) contact with infected patient's skin, mucous membranes, skin lesions, and/or body fluids, for example, sexual contact, contact with patient's saliva to the eye or oral cavity of an uninfected individual, contact with contaminated materials like clothes. b) Being within six feet of an infected patient undergoing procedures that increase the risk of creating aerosol from oral or skin lesions without wearing proper N95 and eye protection, for example, dental procedures, or shaking soiled infected clothes.

Intermediate Risk Exposure

A non-infected individual within six feet of an individual infected with monkeypox without wearing a mask for three hours or more is considered intermediate risk. Individuals should be wearing at least a surgical mask. An additional intermediate degree exposure is when a non-infected person using only gloves and not donning a gown comes in contact with an infected patient's bodily fluids, skin sores, or dirty clothing.

Low-Risk Exposure

CDC considers low risk when a non-infected individual comes in contact with an infected patient a) while taking all proper precautionary measures when entering a patient's room, b) without wearing eye protection regardless of the duration of exposure, and c) is within six feet for less than three hours, and not wearing a mask.

CDC recommends that individuals exposed to the monkeypox virus refrain from donating organs, blood, or breastmilk for at least 21 days to ensure they do not develop symptoms [36].

Post-exposure treatment

Individuals exposed to the monkeypox virus should consult their public health authority, which will determine treatment plans depending on the exposure risk mentioned above. Individuals eligible for prophylactic vaccinations should be vaccinated within four days of exposure to achieve the maximum benefit. Even though the vaccine can be given for up to 14 days post-exposure, if given between four and 14 days post-exposure, it minimizes the symptoms and does not prevent infection [46].

In individuals with high-risk exposure to monkeypox, per CDC guidelines, postexposure modified vaccinia Ankara (MVA) vaccination is required. Immunocompetent individuals may use the ACAM2000 vaccine as well. According to CDC guidelines [46], post-exposure prophylactic vaccination is determined case-by-case for individuals with intermediate risk exposure, including evaluation by likelihood of transmission. Post-exposure prophylactic vaccination is not indicated for individuals with a low risk of exposure.

Vaccinia Immune Globulin

Patients who are immunocompromised and exposed to the monkeypox virus may require the use of vaccinia immune globulin. CDC must be contacted for this agent to be available for the patient if needed.

Post-exposure vaccination

There are two types of vaccination available that can reduce the risk of monkeypox infection.

Modified Vaccinia Ankara (MVA) Vaccine

Sold under the trade name Jynneos (also known as Imvamune or Imvanex) in the United States (US), it is a live, nonreplicating vaccine. This vaccination has an excellent safety profile and can even be used in immunocompromised patients and patients with skin disorders. This vaccine requires two doses subcutaneously, which are administered four weeks apart. This vaccination is made from the nonreplicating vaccinia virus. Common side effects of the MVA vaccine include headache, myalgia, pain at injection site, and lymphadenopathy that last only a few days post-vaccination [47].

ACAM2000

ACAM2000 is a replication-competent smallpox vaccine. This vaccination should be used only in healthy, immunocompetent, and nonpregnant patients with a high risk of exposure. This vaccination is associated with more side effects when compared to the MVA vaccine because ACAM2000 requires an infectious dose on a bifurcated sterile needle that is penetrated into the epidermis of the deltoid region of the arm about 15 times. This process is known as scarification. After two to five days of vaccination, a papule will form, becoming a vesicle after a few more days [48]. The vesicle reaches its maximum size in about one week to 10 days, and then a scab forms in two weeks, which then falls off. Patients vaccinated with ACAM2000 often complain of mild fever and lymphadenopathy for the first two weeks after vaccination.

## Conclusions

The *Orthopoxvirus,* also known as monkeypox virus, was initially discovered in the 1950s. Previously, this virus was primarily confined to Central and West Africa. However, monkeypox virus infection has gradually expanded worldwide with the termination and eradication of the smallpox vaccine and virus. Therefore, on July 23, 2022, the WHO recently designated the monkeypox virus as a public health emergency of international concern (PHEIC). Attributed to the rise in cases, the CDC advises clinicians and other healthcare providers to remain vigilant and report to local health departments if they discover any patients infected with the monkeypox virus. These local health departments can then reach out to the CDC and formulate a strategy for individuals who had direct contact with these patients. Since this infection can spread via droplets or skin-to-skin contact, the healthcare professionals treating a patient suspected of monkeypox should use standard contact and droplet precautions. A crucial lesson is avoiding close contact with suspected patients to reduce transmission. Community education to maximize prevention efforts and destigmatizing to minimize barriers to seeking help are key to containment. 
